# Ethnic disparities in breast cancer survival in New Zealand: which factors contribute?

**DOI:** 10.1186/s12885-017-3797-0

**Published:** 2018-01-08

**Authors:** Sandar Tin Tin, J. Mark Elwood, Charis Brown, Diana Sarfati, Ian Campbell, Nina Scott, Reena Ramsaroop, Sanjeewa Seneviratne, Vernon Harvey, Ross Lawrenson

**Affiliations:** 1 Section of Epidemiology and Biostatistics, School of Population Health, The University of Auckland, Auckland, New Zealand; 2 National Institute of Demographic and Economic Analysis, The University of Waikato, Hamilton, New Zealand; 3 Department of Public Health, The University of Otago, Wellington, New Zealand; 4 Waikato Clinical Campus, The University of Auckland, Hamilton, New Zealand; 5 Waikato District Health Board, Hamilton, New Zealand; 6 Surgical Pathology Department, Waitemata District Health Board, Auckland, New Zealand; 7 Department of Surgery, The University of Colombo, Colombo, Sri Lanka; 8 Auckland District Health Board, Auckland, New Zealand; 9 SMART Marketing and Research, Hamilton, New Zealand

**Keywords:** Breast cancer, Survival, Ethnic groups, Mediation, New Zealand

## Abstract

**Background:**

New Zealand has major ethnic disparities in breast cancer survival with Māori (indigenous people) and Pacific women (immigrants or descended from immigrants from Pacific Islands) faring much worse than other ethnic groups. This paper identified underlying factors and assessed their relative contribution to this risk differential.

**Methods:**

This study involved all women who were diagnosed with primary invasive breast cancer in two health regions, covering about 40% of the national population, between January 2000 and June 2014. Māori and Pacific patients were compared with other ethnic groups in terms of demographics, mode of diagnosis, disease factors and treatment factors. Cox regression modelling was performed with stepwise adjustments, and hazards of excess mortality from breast cancer for Māori and Pacific patients were assessed.

**Results:**

Of the 13,657 patients who were included in this analysis, 1281 (9.4%) were Māori, and 897 (6.6%) were Pacific women. Compared to other ethnic groups, they were younger, more likely to reside in deprived neighbourhoods and to have co-morbidities, and less likely to be diagnosed through screening and with early stage cancer, to be treated in a private care facility, to receive timely cancer treatment, and to receive breast conserving surgery. They had a higher risk of excess mortality from breast cancer (age and year of diagnosis adjusted hazard ratio: 1.76; 95% CI: 1.51–2.04 for Māori and 1.97; 95% CI: 1.67–2.32 for Pacific women), of which 75% and 99% respectively were explained by baseline differences. The most important contributor was late stage at diagnosis. Other contributors included neighbourhood deprivation, mode of diagnosis, type of health care facility where primary cancer treatment was undertaken and type of loco-regional therapy.

**Conclusions:**

Late diagnosis, deprivation and differential access to and quality of cancer care services were the key contributors to ethnic disparities in breast cancer survival in New Zealand. Our findings underscore the need for a greater equity focus along the breast cancer care pathway, with an emphasis on improving access to early diagnosis for Māori and Pacific women.

**Electronic supplementary material:**

The online version of this article (10.1186/s12885-017-3797-0) contains supplementary material, which is available to authorized users.

## Background

Breast cancer is the most common cancer for women worldwide. In New Zealand, breast cancer accounted for almost 30% of all new cancer cases and 14% of all cancer deaths in 2012 [1, 2]. While survival from breast cancer has improved over time, net survival in New Zealand is inferior to some other developed nations [3], including its neighbour Australia [4]. Of great concern is large survival inequities that exist within the country, with the poorest outcomes experienced by Māori (indigenous people, constituting 14% of New Zealand women) and Pacific women (immigrants or descended from immigrants from Pacific Islands, constituting 7% of New Zealand women) [2].

Causes of ethnic disparities in cancer survival are complex and likely to include a range of factors related to patient demographics, tumour biology, and inequities in access, timeliness and quality of care along the cancer diagnosis and treatment pathway. Compared with New Zealand European women, Māori and Pacific women have a younger age distribution [5], and are more likely to live in deprived areas [6], to have comorbid conditions [7], to experience unmet need for health care [8] and to receive poorer quality health care [9].

New Zealand has a publicly funded national health system that provides free secondary health care to all residents but primary health care is only partially government-subsidised. Previous research found that, compared with their New Zealand European counterparts, Māori and Pacific women were more likely to be diagnosed with advanced breast cancer [10], along with being less likely to be diagnosed through screening, [11] and less likely to receive timely and optimal cancer treatment [12–15]. Although differences in tumour biological factors have been postulated as being an important cause of ethnic inequities in breast cancer survival, the evidence to support that hypothesis is weak [16–19]. In previous analyses based on a regional breast cancer register involving 2679 patients, late stage at diagnosis contributed most to survival disparities between Māori and New Zealand European women [20], and differences in biological factors had minimal impact [21]. That study, however, did not investigate disparities in Pacific women. In an earlier study involving 2968 patients identified from the New Zealand Cancer Registry, deprivation and markers of timely access to care (tumour extent and size at presentation) accounted for most of the ethnic disparities in breast cancer survival [22]. The study, however, did not assess the contribution of treatment factors in the analyses.

This study involved 13,657 patients identified from the two regional breast cancer registries and assessed the relative contribution of demographic, disease and treatment factors in explaining survival disparities for Māori, Pacific and non-Māori non-Pacific patients who were diagnosed with primary breast cancer.

## Methods

### Study population

**Fig. 1:**
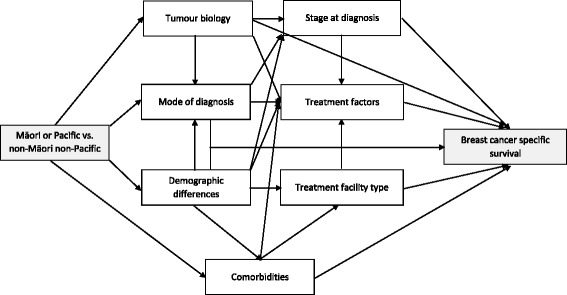
A simplified causal diagram depicting the role of mediating factors

This study involved all women diagnosed with primary invasive breast cancer between January 2000 and June 2014 in the four District Health Board regions (Auckland, Counties Manukau, Waitemata and Waikato), where about two-fifths of the country’s population reside. These combined regions have similar breast cancer incidence and mortality rates to national figures [1].

### Data sources

Participants were identified from the Auckland and Waikato Breast Cancer Registers which are prospectively maintained population-based databases. From 2000 onward, the registers captured almost all newly diagnosed breast cancer cases in the respective district health board regions. Both databases contain much more comprehensive and accurate information than national data sources [23–25].

Using the National Health Index (NHI) number, a unique identifier assigned to every person who uses health and disability support services in New Zealand, the registers are regularly linked to the National Cancer Registry and Mortality Collection. The New Zealand Cancer Registry contains information about all malignant tumours first diagnosed in New Zealand, except basal cell and squamous cell tumours of the skin [26]. The Mortality Collection contains information about all deaths registered in the country [27]. In order to obtain information on comorbidities, Breast Cancer Registry data were also linked to the National Minimum Dataset which contains information about all day patients and inpatients discharged from all public hospitals and over 90% of private hospitals in New Zealand [28].

### Variables of interest

The exposure of interest in this analysis was ethnicity. Patients were classified as Māori, Pacific and non-Māori non-Pacific based on ethnicity recorded in the breast cancer registers. The Waikato Registry collects self-identified ethnicity whereas ethnicity data in the Auckland registry is based on that linked to the National Health Index. Patients with more than one recorded ethnicity were allocated to a single ethnic group in order of priority: Māori, Pacific, Asian and European/Other [29]. The primary study outcome was breast cancer specific mortality. Categorisation of death due to breast cancer was based on medical records and death certificates. Information on cause of death was ascertained by referring to original documents and cross-referencing with other national databases.

Other variables for this analysis were selected based on their likely confounding or mediating effect on the exposure-outcome association, and these include: patients’ demographics such as age and health domicile code, year of cancer diagnosis, mode of diagnosis (screen or symptomatic), tumour characteristics such as stage at diagnosis (Tumour, Node and Metastasis (TNM) system), grade, histological type and hormone receptor status, heath care facility type where primary cancer treatment was undertaken (private or public), time to first treatment, and type of treatment such as loco-regional therapy (i.e., surgery and radiotherapy), chemotherapy and hormonal therapy.

The health domicile codes represent patients’ usual residential address and were categorised as urban (main urban, satellite urban and rural with high urban influence) and rural areas (others) based on Statistics New Zealand’s Urban/Rural Profile [30]. To assess the degree of neighbourhood deprivation, the domicile codes were also mapped on to the 2006 New Zealand Deprivation Index (NZDep) with decile ten the most deprived and decile one the least [31]. Patients’ comorbidity was measured using a C3 index score which is a cancer-specific index of comorbidity based on the presence of 42 chronic conditions recorded in the National Minimum Dataset for a period of 5 years prior to the diagnosis of cancer [32]. Each condition was weighed to its impact on one-year non-cancer mortality in a cancer cohort, and the weights were then summed to get a final comorbidity score.

### Analyses

Analyses involved 13,657 women diagnosed with invasive breast cancer, and excluded 2342 patients with stage 0 or in-situ cancer. Baseline data were presented as means with standard deviations and medians with interquartile ranges for continuous variables and percentages for categorical variables. Missing values were computed using multiple imputation with ten complete datasets created by the Markov chain Monte Carlo method [33], incorporating all baseline co-variables and survival outcomes. Differences in baseline characteristics for Māori, Pacific and non-Māori non-Pacific women were assessed using a two-sample T-test (for continuous variables) and Chi-squared test (for categorical variables) and adjusted for age and the year of diagnosis using PROC MIXED which fits a variety of mixed linear models to the data. Differences in the time to the first treatment and types of cancer treatment received were also adjusted for stage at diagnosis, grade, histological type and hormone receptor status.

Cumulative incidences for breast cancer specific mortality were computed considering death from other causes as the first event as a competing risk. Cox proportional hazards regression modelling was performed with death from breast cancer as the failure variable, and death from another cause, or if alive, date of last follow-up, as censored observations. Hazard ratios (HR) were adjusted for age and year of diagnosis and then sequentially adjusted for seven domains of co-variables: tumour biological factors, area of residence, mode of presentation, stage at diagnosis, comorbidity index, treatment facility type and treatment factors (Figure 1). When the continuous variables (age, time to first treatment and C3 index score) were added to the model, restricted cubic splines were used with knots at the 5th, 50th, and 95th percentiles [34]. HER-2 status was excluded as about one-third of the records had missing values.

The mediating role of each domain was determined by the percentage reduction in the β coefficient after inclusion of each domain in the model using the approach described previously [35]: 100 × (β_crude_-β_adjusted_)/β_crude_. The 95% confidence intervals relating to each percentage attenuation were estimated using a nonparametric bootstrapping method with 2000 re-samplings (with replacement). Subgroup analyses were undertaken by stage at diagnosis (I and II vs. III and IV) and by mode of diagnosis (screen-detected vs. symptomatic). Sensitivity analyses were performed by restricting the sample to patients who were diagnosed from 2006 onward to assess the contribution of HER2, and by using total mortality (deaths from any cause) as the outcome variable. All analyses were performed using SAS (release 9.4, SAS Institute Inc., Cary, North Carolina).

## Results

Of the 13,657 women included in the analysis, 1281 (9.4%) were Māori, and 897 (6.6%) were Pacific women (Table 1). Of the 11,479 (84.0%) non-Māori non-Pacific group, 9679 (84.3%) were New Zealand European, 152 (1.3%) were other European and 1051 (9.2%) were Asian. Compared to the non-Māori non-Pacific group, Māori and Pacific patients were younger, and more likely to reside in deprived neighbourhoods. Māori women were more likely to reside in rural areas and the Waikato region (compared to Auckland) but the opposite was true for Pacific women.

**Table 1:** Baseline characteristics by ethnicity

Characteristics		Māori (N = 1283)		Pacific (N = 898)		Non-Māori Non-Pacific (N = 11,489)		*p*-value	
		Crude	Adjusted	Crude	Adjusted	Crude	Adjusted	Crude	Adjusted
Age	Mean (SD)	54.8 (11.9)		54.1 (12.7)		59.6 (14.0)		<0.0001	
	Median (IQR)	54.0 (16.0)		53.0 (18.0)		58.0 (20.0)			
NZDep 2006									
1–2	%	5.0	*4.7*	4.5	*4.1*	23.4	*24.2*	<0.0001	*<0.0001*
3–4	%	8.3	*8.7*	5.1	*6.4*	18.4	*20.4*		
5–6	%	15.0	*16.2*	10.1	*12.8*	21.0	*23.0*		
7–8	%	23.7	*25.3*	20.5	*23.3*	17.8	*19.0*		
9–10	%	44.4	*45.1*	52.3	*53.5*	13.2	*13.5*		
Missing/unknown	%	*3.7*		*7.5*		*6.2*			
Area of residence									
Urban	%	73.0	*78.3*	85.4	*94.9*	78.3	*86.4*	<0.0001	*<0.0001*
Rural	%	20.1	*21.7*	2.7	*5.1*	12.0	*13.6*		
Missing/unknown	%	*6.9*		*11.9*		*9.7*			
Register									
Auckland	%	63.6	*62.7*	93.5	*92.5*	77.7	*77.9*	<0.0001	*<0.0001* ^*a*^
Waikato	%	36.4	*37.4*	6.5	*7.5*	22.3	*22.1*		
Screen-detected	%	36.1	*35.0*	31.0	*29.9*	39.3	*39.5*	<0.0001	*<0.0001* ^*a*^
<45 years old	%	2.4	*2.8*	0.5	*0.9*	8.2	*8.1*	<0.0001	*<0.0001* ^*a*^
45-69 years old	%	49.7	*49.2*	47.0	*47.2*	54.9	*55.0*	<0.0001	*<0.0001* ^*a*^
70+ years old	%	9.5	*5.2*	7.3	*3.6*	15.5	*15.9*	0.01	*<0.0001* ^*a*^
Stage at diagnosis									
I	%	34.8	*34.9*	25.6	*25.7*	43.6	*43.6*	<0.0001	*<0.0001* ^*a*^
II	%	39.8	*40.3*	40.5	*41.0*	38.4	*38.4*		
III	%	17.9	*17.3*	23.2	*22.4*	13.9	*14.1*		
IV	%	7.3	*7.55*	10.6	*10.9*	3.9	*3.9*		
*Missing/unknown*	%	*0.1*		*0.1*		*0.2*			
Grade									
I	%	20.6	*21.8*	15.7	*17.2*	24.1	*24.7*	<0.0001	*<0.0001* ^*a*^
II	%	46.7	*51.1*	42.8	*47.9*	44.0	*46.9*		
III	%	26.9	*27.1*	35.0	*34.9*	26.8	*28.4*		
*Missing/unknown*	%	*5.8*		*6.6*		*5.1*			
Histology									
Ductal	%	83.6	*82.8*	83.9	*83.0*	78.8	*79.5*	<0.0001	*0.2* ^*a*^
Lobular	%	7.8	*8.9*	6.5	*7.9*	12.1	*12.5*		
Other	%	7.9	*8.3*	8.6	*9.1*	8.0	*8.0*		
Missing/unknown	%	*0.8*		*1.1*		*1.1*			
Hormone receptor status									
ER+/PR+	%	67.7	*67.8*	65.6	*65.8*	63.2	*63.9*	<0.0001	*0.03* ^*a*^
ER+/PR-	%	11.9	*14.0*	8.1	*11.5*	14.8	*15.9*		
ER−/PR+	%	1.4	*2.3*	1.3	*2.5*	1.4	*2.2*		
ER−/PR-	%	16.3	*15.9*	20.7	*20.2*	17.8	*18.1*		
Missing/unknown	%	*2.7*		*4.2*		*2.8*			
HER-2									
Positive	%	16.1	*16.2*	20.8	*21.3*	11.8	*13.6*	<0.0001	*<0.0001* ^*a*^
Equivocal	%	1.8	*13.6*	1.0	*15.3*	1.7	*13.2*		
Negative	%	65.6	*70.3*	56.4	*63.4*	64.0	*73.3*		
Missing/unknown	%	*16.5*		*21.8*		*22.5*			
C3 index scores									
0	%	69.8	*65.6*	72.4	*67.5*	79.5	*80.3*	<0.0001	*<0.0001* ^*a*^
1	%	9.6	*10.3*	8.4	*9.2*	7.9	*7.7*		
2	%	8.6	*24.1*	7.8	*23.3*	5.3	*11.9*		
Treatment facility type									
Public	%	83.8	*86.6*	84.2	*87.3*	53.2	*52.6*	<0.0001	*<0.0001* ^*a*^
Private	%	16.2	*13.4*	15.8	*12.7*	46.8	*47.4*		
Time to first treatment (days)^b^	Mean (SD)	41.2 (54.9)	*41.5*	62.5 (154.6)	*62.1*	30.4 (54.9)	*30.3*	<0.0001	*<0.0001* ^*c*^
	Median (IQR)	33.0 (25.0)		34.0 (30.0)		23.0 (23.0)			
Loco-regional therapy									
Breast conserving surgery + radiotherapy	%	37.4	*38.2*	27.2	*32.1*	42.7	*42.2*	<0.0001	*<0.0001* ^*c*^
Breast conserving surgery alone	%	7.4	*8.1*	6.4	*8.0*	9.1	*8.9*		
Mastectomy + radiotherapy	%	20.8	*17.6*	23.5	*16.4*	16.4	*17.3*		
Mastectomy alone	%	25.9	*27.7*	28.6	*31.1*	25.7	*25.3*		
No primary surgery	%	8.5	*8.4*	14.4	*12.4*	6.1	*6.3*		
Systemic therapy									
Chemotherapy + hormonal/biological treatment	%	27.5	*20.9*	26.8	*16.8*	22.3	*23.8*	<0.0001	*<0.0001* ^*c*^
Chemotherapy alone	%	10.9	*10.7*	9.0	*6.3*	9.6	*9.9*		
Hormonal/biological treatment alone	%	39.7	*42.6*	38.9	*44.8*	40.6	*39.8*		
None	%	21.9	*25.92*	25.3	*32.1*	27.5	*26.5*		

^a^Missing data imputed and proportion adjusted for age and year of diagnosis

^b^Restricted to 13,314 patients who received cancer treatment during follow-up

^c^Missing data imputed and proportion adjusted for age, year of diagnosis and disease factors (stage at diagnosis, grade, histological type and ER/PR status)

Compared with the non-Māori non-Pacific group, Māori and Pacific women were less likely to be diagnosed through screening and with stage 1 and grade 1 cancer and more likely to have ductal cancer compared to other ethnic groups. Māori women were more likely to be oestrogen and progesterone receptor positive. Both Māori and Pacific women were more likely to have comorbidities. They were less likely to be treated in a private care facility, had a significantly longer time to first treatment after diagnosis, and were less likely to receive breast conserving surgery even after adjusting for stage at diagnosis. Pacific patients were also less likely to receive chemotherapy.

**Fig. 2:**
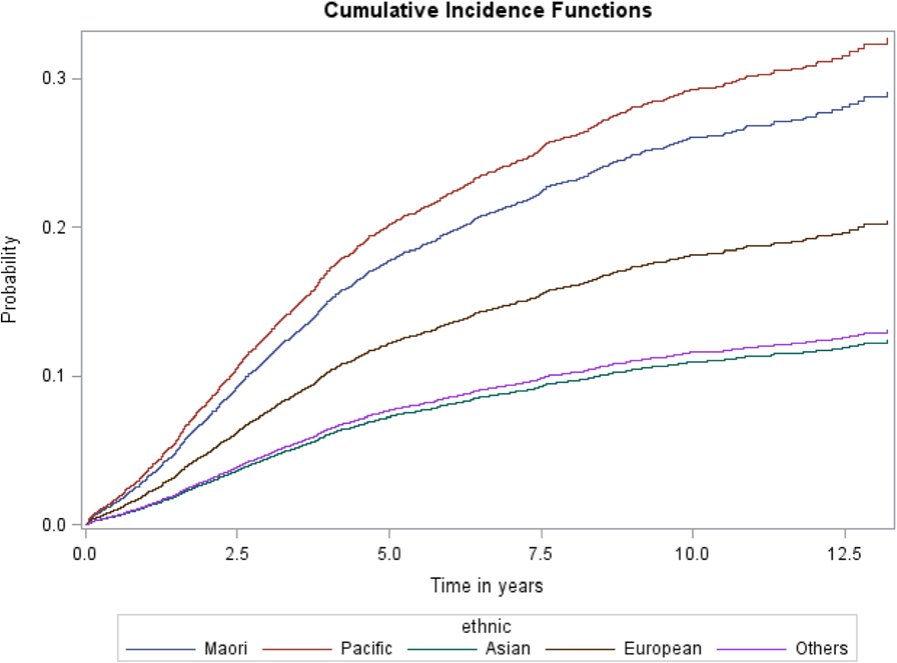
Cumulative breast cancer specific mortality by ethnicity

During a median follow-up of 4.2 years, Māori women had a significantly higher risk of mortality from breast cancer than non-Māori non-Pacific women (age and year of diagnosis adjusted HR 1.76; 95% CI: 1.51, 2.04) (Fig. 2 and Table 2). Adjustments for residential factors, mode of diagnosis, disease factors, C3 index score, treatment facility type and treatment factors resulted in a 75% reduction in the HR. In particular, neighbourhood deprivation, mode of presentation, stage at diagnosis, and type of loco-regional therapy contributed most to the risk differential between Māori and non-Māori non-Pacific patients. After adjustment for all factors, the risk of mortality was still 15% higher in Māori patients (HR 1.15; 0.97–1.37).

**Table 2:** Hazards of death from breast cancer in Māori and Pacific women in comparison with non-Māori non-Pacific women

Models	Additional variables in the model	Hazard ratios (95% CI)	% attenuation^a^ (95% CI^b^)
Māori vs. non-Māori non-Pacific			
1. Age and year of diagnosis adjusted		1.76 (1.51, 2.04)	
2. Model 1 + Tumour biology	Grade	1.73 (1.49, 2.01)	
	Histology	1.73 (1.49, 2.02)	
	Hormone receptor status	1.82 (1.56, 2.13)	−6.5 (−18.3, 2.3)
3. Model 2 + Area of residence	NZDep2006	1.70 (1.45, 1.99)	
	Rurality	1.69 (1.44, 1.98)	
	Registries	1.67 (1.42, 1.96)	9.2 (−3.7, 22.6)
4. Model 3 + Mode of diagnosis	Screen detected	1.58 (1.35, 1.85)	18.7 (5.7, 33.7)
5. Model 4 + Tumour stage at diagnosis	Stage	1.26 (1.07, 1.49)	58.8 (33.9, 83.1)
6. Model 5 + Comorbidity	C3 index scores	1.27 (1.07, 1.49)	58.2 (32.6, 82.0)
7. Model 6 + Treatment facility type	Public vs. private	1.21 (1.02, 1.43)	66.5 (44.0, 97.6)
8. Model 7 + Treatment factors	Time to first treatment	1.21 (1.02, 1.43)	
	Loco-regional therapy	1.14 (0.95, 1.36)	
	Systemic therapy	1.15 (0.97, 1.37)	74.9 (51.2, 110.0)
Pacific vs. non-Māori non-Pacific			
1. Age and year of diagnosis adjusted		1.97 (1.67, 2.32)	
2. Model 1 + Tumour biology	Grade	1.78 (1.50, 2.10)	
	Histology	1.78 (1.50, 2.11)	8.9 (−0.6, 18.2)
	Hormone receptor status	1.85 (1.56, 2.20)	
3. Model 2 + Area of residence	NZDep2006	1.70 (1.42, 2.03)	
	Rurality	1.71 (1.43, 2.05)	
	Registries	1.78 (1.48, 2.13)	15.0 (0.2, 30.2)
4. Model 3 + Mode of diagnosis	Screen detected	1.66 (1.38, 1.99)	25.0 (10.4, 42.1)
5. Model 4 + Tumour stage at diagnosis	Stage	1.19 (0.98, 1.44)	74.3 (55.8, 106.8)
6. Model 5 + Comorbidity	C3 index scores	1.19 (0.98, 1.44)	74.5 (55.8, 106.8)
7. Model 6 + Treatment facility type	Public vs. private	1.11 (0.92, 1.35)	84.0 (62.9, 117.6)
8. Model 7 + Treatment factors	Time to first treatment	1.12 (0.92, 1.36)	
	Loco-regional therapy	1.04 (0.85, 1.27)	
	Systemic therapy	1.01 (0.82, 1.23)	98.9 (74.1, 137.6)

^a^% attenuation compared with Model 1

^b^95% bootstrap confidence interval

Pacific women had a significantly higher risk of mortality from breast cancer than non-Māori non-Pacific women (age and year of diagnosis adjusted HR 1.97; 95% CI: 1.67, 2.32) (Fig. 2 and Table 2). Factors included in the fully adjusted model reduced the HR to 1.01 (95% CI: 0.82, 1.23). Tumour grade, neighbourhood deprivation, mode of presentation, stage at diagnosis, and type of loco-regional therapy contributed most to the risk difference.

In subgroup analyses, the associations were similar in early vs. advanced breast cancer (Additional file 1: Table S1) but were weaker in women who were diagnosed through screening (Additional file 1: Table S2). In sensitivity analyses restricted to patients who were diagnosed from 2006 onward, ethnic differences in HER2 status contributed modestly to survival disparities in both Māori and Pacific women (Additional file 1: Table S3). When total mortality was used as the failure variable, similar but stronger associations were observed (Additional file 1: Table S4). The higher C3 index score also contributed to ethnic disparities in overall survival.

## Discussion

### Main findings

This paper confirmed that survival disparities exist for both Māori and Pacific women in the New Zealand context. Our work shows that there are differences between Māori and Pacific and non-Māori non-Pacific women groups in terms of residential area, mode of presentation, disease factors, comorbidity index, treatment factors and cancer care facility type. These differences explained approximately 75% and 99% respectively of the survival disparities between Māori and Pacific and non-Māori non-Pacific women. The most important contributor was late stage at diagnosis. Other contributors included neighbourhood deprivation, mode of presentation, treatment facility type and type of loco-regional therapy. Tumour grade also contributed to the survival differential in Pacific women.

### Interpretations

Māori and Pacific women were almost twice as likely to die from breast cancer as non-Māori non-Pacific women. This is consistent with the findings from previous New Zealand research [19, 20, 22, 36–38]. The findings also contribute to a growing literature on ethnic/racial disparities in breast cancer incidence and outcomes internationally [39–42].

Such disparities are likely to be due to social, biological and health system determinants of poor outcomes [42]. Consistent with previous research in New Zealand and elsewhere [20, 43], stage at diagnosis accounted for a substantial proportion of the survival differential in Māori and Pacific women. The high prevalence of late diagnosis in both ethnic groups could be partly explained by unequal screening coverage. New Zealand has a publicly funded national breast cancer mammography screening program for asymptomatic women aged 50 to 64 years since 1998. The program was extended to include women aged 45 to 49 years and 65 to 69 years in 2004. While total population screening coverage has improved over time and met the target of 70%, coverage for Māori women remains relatively low (65% nationally and 60% and 59% respectively in Auckland and Waikato) [44]. The national coverage for Pacific women has exceeded the target (72%) but the coverage is still low in some regions including the Waikato at 63%. We found that Pacific women in the screening age group were least likely to be diagnosed through screening (47% in Pacific, 50% in Māori and 55% in non-Māori non-Pacific women). Other factors that could contribute to late diagnosis include greater barriers related to access to primary care practices and practitioners, low health literacy, low socioeconomic status and psychosocial factors [45–48]. These are worthy of further investigation particularly because the differential stage distribution was also a key contributor to survival disparities in symptomatic patients.

In New Zealand, socioeconomic position has been reported to be an important determinant of health and a mediator in the association between ethnicity and health [49]. In this study, about half of Māori and Pacific women resided in the most deprived neighbourhoods (cf. 13% of non-Māori non-Pacific women). While NZDep2006 measures area-level deprivation and may not reflect an individual’s actual socioeconomic status, it may be regarded as a marker of health care access. In a recent population-based case-control study involving patients with breast cancer, Māori and Pacific women were more likely to report “cost” as a barrier to accessing cancer care [50]. Many studies have also linked aspects of socioeconomic deprivation with poorer cancer survival through late diagnosis and differential treatment [51, 52].

Māori and Pacific women (16% each) were significantly less likely to access private care for their primary treatment for breast cancer than non-Māori non-Pacific women (47%). In New Zealand, alongside the publicly funded national health system, the private system provides a range of services including elective treatments and general surgical procedures which are mostly funded by private health insurance. Our previous research has linked private care with earlier diagnoses, better treatments and higher survival from breast cancer [53]. In this analysis, we found that differential access to private care accounted for 8% and 10% respectively of survival disparities in Māori and Pacific women.

In addition to experiencing diagnostic delays, Māori and Pacific women had a significantly longer time to the first treatment for breast cancer as reported previously [54]; however, treatment delays following diagnosis contributed minimally to the survival inequities between ethnic groups. This throws into question, the usefulness of the current New Zealand Ministry of Health “Faster Cancer Treatment (FCT) Programme” for rectifying the disparities in mortality we have shown. The FCT programme aims to reduce waiting time for appointments, tests and treatment and sets a 62-day cancer treatment target [55], and was adopted from the very similar UK National Health Service programme. Our analysis shows that it is the delay to the initial diagnosis rather than delay to the start of treatment following diagnosis that has contributed to the poorer outcome in Māori and Pacific women. This indicates that more effort is required to improve early diagnosis of breast cancer for Māori and Pacific women. A priority must be to increasing access to the National Screening Programme for these women. Other possible actions include enhancing access to primary care for Māori and Pacific women with breast lumps, improving transition between primary and secondary care, raising awareness of the disease and emphasising the importance of early diagnosis.

We found that the type of loco-regional therapy also contributed to ethnic disparities. Similar to what has been observed in the US black vs. white populations [56], Māori and Pacific women were less likely to receive primary surgery and even when they did, they were less likely to receive breast conserving surgery and radiotherapy. Our analysis accounted for stage at diagnosis and biological factors, so this does not reflect a higher prevalence of advanced cancer in Māori and Pacific women. Another possible explanation is the fact that Māori and Pacific women are more likely to be treated in the public sector where the rate of primary surgery and radiotherapy appears to be lower [53, 57, 58].

### Strengths and limitations

This study used data from the two prospectively maintained population-based databases which contain comprehensive and near complete information about patients diagnosed with primary breast cancer. Linkage to national databases enabled us to ascertain information on cause of death and to obtain information on comorbidities. However, potential misclassification of cancer-specific deaths may still occur and may reduce observed differences to a small extent. Information on ethnicity recorded in the Auckland registry may not be accurate as it is based on that linked to the National Health Index. Comparisons between such data and census data have shown undercounting of Māori by 15% and of Pacific people by 10% [59]. This may move the observed risk estimates toward the null, but in mediation analyses, misclassification of the mediating variables is more important than that of the exposure and may underestimate the % attenuation presented in this paper [60]. As a considerable number of patients had missing data on HER-2 status - most of whom were diagnosed prior to 2006 when HER-2 testing was not routine in New Zealand, we excluded HER-2 status from the analyses. The contribution of HER2 was minimal in sensitivity analyses restricted to patients who were diagnosed from 2006 onward. It was not possible to assess the impact of some important factors such as body mass index and smoking, as relevant data were not recorded in the databases.

## Conclusions

Māori and Pacific women had a higher risk of mortality from breast cancer compared to other ethnic groups. This was largely contributed by late stage at diagnosis and also by important differences in neighbourhood deprivation, mode of diagnosis, treatment facility type and type of loco-regional therapy. Our findings underscore the need for a greater equity focus along the breast cancer care pathway in New Zealand, with an emphasis on improving access to early diagnosis for Māori and Pacific women. This may result in greater impact on improving outcomes for women than just focusing on faster treatment once diagnosed.

## Additional file

Additional file 1: Table S1. Hazards of death from breast cancer in Māori and Pacific women in comparison with non-Māori non-Pacific women by tumour stage at diagnosis. **Table S2.** Hazards of death from breast cancer in Māori and Pacific women in comparison with non-Māori non-Pacific women by mode of diagnosis. **Table S3.** Hazards of death from breast cancer in Māori and Pacific women in comparison with non-Māori non-Pacific women who were diagnosed from 2006 onward. **Table S4.**. Hazards of death from all causes in Māori and Pacific women in comparison with non-Māori non-Pacific women (DOCX 22 kb)

## Abbreviations

CI: Confidence Interval
ER: Oestrogen Receptor
FCT: Faster Cancer Treatment
HER-2: Human Epidermal Growth Factor Receptor 2
HR: Hazard Ratio
NHI: National Health Index
NZDep: New Zealand Deprivation Index
PR: Progesterone Receptor
