# Supplementary material for: Ethnic disparities in breast cancer survival in New Zealand: which factors contribute?
Source: BMC Cancer. 2018 Jan 8;18:58. doi: 10.1186/s12885-017-3797-0 (PMC5759270; doi:10.1186/s12885-017-3797-0)
Supplement: Additional file 1: Table S1. — Hazards of death from breast cancer in Māori and Pacific women in comparison with non-Māori non-Pacific women by tumour stage at diagnosis. Table S2. Hazards of death from breast cancer in Māori and Pacific women in comparison with non-Māori non-Pacific women by mode of diagnosis. Table S3. Hazards of death from breast cancer in Māori and Pacific women in comparison with non-Māori non-Pacific women who were diagnosed from 2006 onward. Table S4.. Hazards of death from all causes in Māori and Pacific women in comparison with non-Māori non-Pacific women (DOCX 22 kb) [file 12885_2017_3797_MOESM1_ESM.docx]

**Table S1. Hazards of death from breast cancer in Māori and Pacific women in comparison with non-Māori non-Pacific women by tumour stage at diagnosis**

| **Models** | **Additional variables in the model** | **Stages I and II** | | **Stages III and IV** | |
| --- | --- | --- | --- | --- | --- |
|  |  | **Hazard ratios (95% CI)** | **% attenuation^a^** | **Hazard ratios (95% CI)** | **% attenuation^a^** |
| ***Māori vs. non-Māori non-Pacific*** |  |  |  |  |  |
| 1. Age and year of diagnosis adjusted |  | 1.35 (1.03, 1.78) |  | 1.45 (1.21, 1.73) |  |
| 2. Model 1 + Tumour biology | Grade | 1.38 (1.05, 1.81) |  | 1.42 (1.18, 1.71) |  |
|  | Histology | 1.37 (1.04, 1.81) |  | 1.41 (1.17, 1.70) |  |
|  | Hormone receptor status | 1.45 (1.10, 1.91) | -21.9 | 1.46 (1.21, 1.76) | -1.6 |
| 3. Model 2 + Area of residence | NZDep2006 | 1.40 (1.05, 1.86) |  | 1.42 (1.17, 1.73) |  |
|  | Rurality | 1.38 (1.04, 1.84) |  | 1.43 (1.17, 1.73) |  |
|  | Registries | 1.37 (1.03, 1.83) | -3.8 | 1.41 (1.16, 1.71) | 7.8 |
| 4. Model 3 + Mode of diagnosis | Screen detected | 1.32 (0.99, 1.76) | 8.4 | 1.36 (1.12, 1.66) | 16.0 |
| 5. Model 4 + Tumour stage at diagnosis | Stage | 1.26 (0.95, 1.68) | 23.6 | 1.24 (1.02, 1.51) | 41.9 |
| 6. Model 5 + Comorbidity | C3 index scores | 1.27 (0.95, 1.70) | 20.7 | 1.25 (1.03, 1.53) | 39.1 |
| 7. Model 6 + Treatment facility type | Public vs. private | 1.24 (0.93, 1.66) | 28.8 | 1.18 (0.97, 1.45) | 54.7 |
| 8. Model 7 + Treatment factors | Time to first treatment | 1.24 (0.93, 1.66) |  | 1.18 (0.96, 1.45) |  |
|  | Loco-regional therapy | 1.22 (0.91, 1.63) |  | 1.09 (0.88, 1.35) |  |
|  | Systemic therapy | 1.21 (0.90, 1.62) | 37.0 | 1.14 (0.92, 1.42) | 64.5 |
| ***Pacific vs. non-Māori non-Pacific*** |  |  |  |  |  |
| 1. Age and year of diagnosis adjusted |  | 1.53 (1.13, 2.07) |  | 1.41 (1.16, 1.71) |  |
| 2. Model 1 + Tumour biology | Grade | 1.37 (1.00, 1.86) |  | 1.40 (1.16, 1.70) |  |
|  | Histology | 1.35 (0.99, 1.85) |  | 1.40 (1.16, 1.70) |  |
|  | Hormone receptor status | 1.41 (1.03, 1.92) | 19.0 | 1.45 (1.18, 1.77) | -7.1 |
| 3. Model 2 + Area of residence | NZDep2006 | 1.30 (0.94, 1.79) |  | 1.40 (1.13, 1.73) |  |
|  | Rurality | 1.33 (0.96, 1.83) |  | 1.38 (1.11, 1.71) |  |
|  | Registries | 1.39 (1.01, 1.93) | 21.5 | 1.40 (1.12, 1.73) | 3.2 |
| 4. Model 3 + Mode of diagnosis | Screen detected | 1.33 (0.97, 1.85) | 31.7 | 1.34 (1.08, 1.67) | 14.3 |
| 5. Model 4 + Tumour stage at diagnosis | Stage | 1.19 (0.86, 1.65) | 58.4 | 1.15 (0.91, 1.45) | 59.0 |
| 6. Model 5 + Comorbidity | C3 index scores | 1.19 (0.86, 1.66) | 58.4 | 1.15 (0.91, 1.45) | 59.4 |
| 7. Model 6 + Treatment facility type | Public vs. private | 1.15 (0.83, 1.61) | 66.7 | 1.06 (0.84, 1.34) | 83.6 |
| 8. Model 7 + Treatment factors | Time to first treatment | 1.14 (0.82, 1.58) |  | 1.07 (0.84, 1.35) |  |
|  | Loco-regional therapy | 1.08 (0.78, 1.51) |  | 0.96 (0.75, 1.23) |  |
|  | Systemic therapy | 1.06 (0.76, 1.48) | 86.1 | 0.94 (0.72, 1.21) | 119.0 |

a % attenuation compared with Model 1

**Table S2. Hazards of death from breast cancer in Māori and Pacific women in comparison with non-Māori non-Pacific women by mode of diagnosis**

| **Models** | **Additional variables in the model** | **Screen detected** | | **Symptomatic** | |
| --- | --- | --- | --- | --- | --- |
|  |  | **Hazard ratios (95% CI)** | **% attenuation^a^** | **Hazard ratios (95% CI)** | **% attenuation^a^** |
| ***Māori vs. non-Māori non-Pacific*** |  |  |  |  |  |
| 1. Age and year of diagnosis adjusted |  | 1.23 (0.73, 2.08) |  | 1.66 (1.42, 1.95) |  |
| 2. Model 1 + Tumour biology | Grade | 1.20 (0.71, 2.05) |  | 1.68 (1.44, 1.97) |  |
|  | Histology | 1.20 (0.71, 2.05) |  | 1.69 (1.44, 1.98) |  |
|  | Hormone receptor status | 1.34 (0.79, 2.28) | -39.9 | 1.76 (1.50, 2.07) | -11.1 |
| 3. Model 2 + Area of residence | NZDep2006 | 1.20 (0.69, 2.11) |  | 1.66 (1.41, 1.96) |  |
|  | Rurality | 1.19 (0.68, 2.08) |  | 1.65 (1.40, 1.95) |  |
|  | Registries | 1.17 (0.67, 2.05) | 23.7 | 1.63 (1.38, 1.93) | 3.8 |
| 4. Model 3 + Tumour stage at diagnosis | Stage | 0.98 (0.55, 1.74) | 109.3 | 1.29 (1.09, 1.54) | 49.5 |
| 5. Model 4 + Comorbidity | C3 index scores | 0.90 (0.50, 1.63) | 150.8 | 1.30 (1.10, 1.55) | 47.9 |
| 6. Model 5 + Treatment facility type | Public vs. private | 0.87 (0.48, 1.58) | 168.6 | 1.24 (1.04, 1.48) | 57.7 |
| 7. Model 6 + Treatment factors | Time to first treatment | 0.90 (0.49, 1.65) |  | 1.24 (1.04, 1.48) |  |
|  | Loco-regional therapy | 0.90 (0.48, 1.71) |  | 1.16 (0.97, 1.39) |  |
|  | Systemic therapy | 0.95 (0.51, 1.80) | 122.3 | 1.17 (0.98, 1.40) | 69.3 |
| ***Pacific vs. non-Māori non-Pacific*** |  |  |  |  |  |
| 1. Age and year of diagnosis adjusted |  | 1.19 (0.61, 2.35) |  | 1.82 (1.53, 2.15) |  |
| 2. Model 1 + Tumour biology | Grade | 1.09 (0.55, 2.16) |  | 1.71 (1.44, 2.04) |  |
|  | Histology | 1.09 (0.55, 2.17) |  | 1.72 (1.45, 2.05) |  |
|  | Hormone receptor status | 1.23 (0.61, 2.45) | -15.4 | 1.77 (1.48, 2.11) | 4.5 |
| 3. Model 2 + Area of residence | NZDep2006 | 1.04 (0.52, 2.08) |  | 1.65 (1.37, 1.99) |  |
|  | Rurality | 1.07 (0.53, 2.17) |  | 1.66 (1.38, 2.01) |  |
|  | Registries | 1.14 (0.57, 2.31) | 24.0 | 1.72 (1.43, 2.08) | 9.2 |
| 4. Model 3 + Tumour stage at diagnosis | Stage | 0.86 (0.38, 1.93) | 187.9 | 1.22 (1.00, 1.49) | 66.8 |
| 5. Model 4 + Comorbidity | C3 index scores | 0.80 (0.36, 1.77) | 224.0 | 1.22 (1.00, 1.49) | 66.7 |
| 6. Model 5 + Treatment facility type | Public vs. private | 0.74 (0.33, 1.64) | 271.9 | 1.14 (0.94, 1.40) | 77.3 |
| 7. Model 6 + Treatment factors | Time to first treatment | 0.73 (0.33, 1.64) |  | 1.15 (0.94, 1.41) |  |
|  | Loco-regional therapy | 0.64 (0.29, 1.44) |  | 1.07 (0.87, 1.32) |  |
|  | Systemic therapy | 0.62 (0.27, 1.42) | 369.5 | 1.03 (0.84, 1.27) | 94.4 |

a % attenuation compared with Model 1

**Table S2. Hazards of death from breast cancer in Māori and Pacific women in comparison with non-Māori non-Pacific women who were diagnosed from 2006 onward**

| **Models** | **Additional variables in the model** | **Hazard ratios (95% CI)** | **% attenuation^a^** |
| --- | --- | --- | --- |
| ***Māori vs. non-Māori non-Pacific*** |  |  |  |
| 1. Age and year of diagnosis adjusted |  | 1.94 (1.55, 2.41) |  |
| 2. Model 1 + Tumour biology | Grade | 1.95 (1.57, 2.43) |  |
|  | Histology | 1.95 (1.57, 2.43) |  |
|  | Hormone receptor status | 2.07 (1.66, 2.58) | -10.1 |
|  | HER2 status | 2.05 (1.64, 2.56) |  |
| 3. Model 2 + Area of residence | NZDep2006 | 1.81 (1.43, 2.27) |  |
|  | Rurality | 1.79 (1.43, 2.26) |  |
|  | Registries | 1.74 (1.38, 2.19) | 16.2 |
| 4. Model 3 + Mode of diagnosis | Screen detected | 1.71 (1.36, 2.15) | 18.8 |
| 5. Model 4 + Tumour stage at diagnosis | Stage | 1.41 (1.10, 1.80) | 48.3 |
| 6. Model 5 + Comorbidity | C3 index scores | 1.40 (1.10, 1.79) | 48.6 |
| 7. Model 6 + Treatment facility type | Public vs. private | 1.31 (1.03, 1.68) | 58.7 |
| 8. Model 7 + Treatment factors | Time to first treatment | 1.30 (1.02, 1.67) |  |
|  | Loco-regional therapy | 1.15 (0.88, 1.50) |  |
|  | Systemic therapy | 1.12 (0.06, 21.16) | 83.0 |
| ***Pacific vs. non-Māori non-Pacific*** |  |  |  |
| 1. Age and year of diagnosis adjusted |  | 2.11 (1.65, 2.70) |  |
| 2. Model 1 + Tumour biology | Grade | 1.94 (1.52, 2.48) |  |
|  | Histology | 1.94 (1.52, 2.48) |  |
|  | Hormone receptor status | 2.08 (1.63, 2.67) | 1.6 |
|  | HER2 status | 2.05 (1.60, 2.63) |  |
| 3. Model 2 + Area of residence | NZDep2006 | 1.77 (1.36, 2.30) |  |
|  | Rurality | 1.81 (1.39, 2.36) |  |
|  | Registries | 1.92 (1.47, 2.51) | 12.7 |
| 4. Model 3 + Mode of diagnosis | Screen detected | 1.89 (1.44, 2.47) | 15.0 |
| 5. Model 4 + Tumour stage at diagnosis | Stage | 1.37 (1.04, 1.80) | 58.2 |
| 6. Model 5 + Comorbidity | C3 index scores |  | 54.5 |
| 7. Model 6 + Treatment facility type | Public vs. private | 1.25 (0.25, 6.17) | 70.4 |
| 8. Model 7 + Treatment factors | Time to first treatment | 1.25 (0.92, 1.70) |  |
|  | Loco-regional therapy | 0.99 (0.00, ∞) |  |
|  | Systemic therapy | 0.92 (0.00, ∞) | 110.6 |

**Table S4. Hazards of death from all causes in Māori and Pacific women in comparison with non-Māori non-Pacific women**

| **Models** | **Additional variables in the model** | **Hazard ratios (95% CI)** | **% attenuation^a^** |
| --- | --- | --- | --- |
| ***Māori vs. non-Māori non-Pacific*** |  |  |  |
| 1. Age and year of diagnosis adjusted |  | 2.14 (1.90, 2.41) |  |
| 2. Model 1 + Tumour biology | Grade | 2.15 (1.90, 2.42) |  |
|  | Histology | 2.15 (1.91, 2.43) |  |
|  | Hormone receptor status | 2.23 (1.98, 2.53) | -5.6 |
| 3. Model 2 + Area of residence | NZDep2006 | 2.12 (1.87, 2.41) |  |
|  | Rurality | 2.11 (1.86, 2.40) |  |
|  | Registries | 2.10 (1.85, 2.38) | 2.7 |
| 4. Model 3 + Mode of diagnosis | Screen detected | 2.02 (1.78, 2.29) | 7.6 |
| 5. Model 4 + Tumour stage at diagnosis | Stage | 1.83 (1.61, 2.08) | 20.8 |
| 6. Model 5 + Comorbidity | C3 index scores | 1.70 (1.50, 1.94) | 30.0 |
| 7. Model 6 + Treatment facility type | Public vs. private | 1.62 (1.42, 1.84) | 37.0 |
| 8. Model 7 + Treatment factors | Time to first treatment | 1.62 (1.43, 1.85) |  |
|  | Loco-regional therapy | 1.53 (1.33, 1.75) |  |
|  | Systemic therapy | 1.54 (1.34, 1.76) | 43.7 |
| ***Pacific vs. non-Māori non-Pacific*** |  |  |  |
| 1. Age and year of diagnosis adjusted |  | 2.07 (1.79, 2.39) |  |
| 2. Model 1 + Tumour biology | Grade | 1.91 (1.63, 2.24) |  |
|  | Histology | 1.92 (1.64, 2.25) |  |
|  | Hormone receptor status | 1.99 (1.70, 2.33) | 5.3 |
| 3. Model 2 + Area of residence | NZDep2006 | 1.87 (1.60, 2.20) |  |
|  | Rurality | 1.90 (1.62, 2.24) |  |
|  | Registries | 1.95 (1.66, 2.30) | 7.9 |
| 4. Model 3 + Mode of diagnosis | Screen detected | 1.85 (1.57, 2.18) | 15.3 |
| 5. Model 4 + Tumour stage at diagnosis | Stage | 1.51 (1.28, 1.78) | 43.2 |
| 6. Model 5 + Comorbidity | C3 index scores | 1.45 (1.23, 1.72) | 48.6 |
| 7. Model 6 + Treatment facility type | Public vs. private | 1.36 (1.14, 1.61) | 58.1 |
| 8. Model 7 + Treatment factors | Time to first treatment | 1.35 (1.14, 1.60) |  |
|  | Loco-regional therapy | 1.31 (1.12, 1.53) |  |
|  | Systemic therapy | 1.27 (1.08, 1.49) | 66.9 |

a % attenuation compared with Model 1
